# Strategies Used to Improve the Performance of Plant Growth-Promoting Rhizobacteria Under Agricultural Greenhouse and Field Production Scenarios in Semi-Arid Conditions

**DOI:** 10.3390/plants15203185

**Published:** 2026-10-17

**Authors:** José Humberto Valenzuela-Soto, Adolfo Baylón-Palomino, Jorge Luis Méndez-Hernández, Alberto Flores-Olivas, John Paul Délano-Frier

**Affiliations:** 1 SECIHTI-Centro de Investigación en Química Aplicada, Blvd. Enrique Reyna 104, Saltillo 25294, Coahuila, Mexico; humberto.valenzuela@ciqa.edu.mx; 2 Centro de Investigación en Química Aplicada, Blvd. Enrique Reyna 104, Saltillo 25294, Coahuila, Mexico; adolfo.baylon@ciqa.edu.mx (A.B.-P.); jorge.mendez.ma24@ciqa.edu.mx (J.L.M.-H.); 3 Departamento de Parasitología, Buenavista, Universidad Autónoma Agraria Antonio Narro, Saltillo 25315, Coahuila, Mexico; aflooli@uaaan.mx; 4 Centro de Investigación y de Estudios Avanzados, Unidad Irapuato, Km 9.6 Libramiento Norte, Irapuato 36821, Guanajuato, Mexico

**Keywords:** PGPR, solanaceous crops, field trials, shading, mulching, crop yields, root exudates, induced systemic resistance, semi-arid agriculture

## Abstract

The utilization of plant growth-promoting rhizobacteria (PGPR) in agricultural production scenarios has the potential to significantly improve the quality and yield of several commercially important crops. The observed benefits stem from the capacity that PGPRs have to alter the biochemistry of their plant hosts to promote growth and/or resist abiotic stress conditions or biotic aggressors. However, a growing body of experimental evidence has demonstrated that the performance of these beneficial microorganisms in the frequently unpredictable real-world conditions of agricultural production is far from satisfactory. Several aspects, from unrealistic laboratory conditions to inefficient distribution networks, contribute to impede the realization of the full potential that PGPRs have to significantly increase production in a much more sustainable manner. The present assay is a compilation of different strategies that have been tested to improve the performance of environmentally resilient PGPRs isolated from the semi-desertic regions of the North–Central state of Coahuila, Mexico, in actual agricultural production scenarios of solanaceous crops, like tomato, chili peppers and tobacco, as well as short-cycle parsley plants. The latter included cultivation in open fields, in addition to the use of protective shading combined, or not, with plastic mulching. The outcome of these working strategies is described, including an ample discussion offering possible explanations for both the positive results and the non-reproducible and/or unexpected poor outcomes. A forward leap into future perspectives to improve the sustainability of the world’s agricultural production is also included.

## 1. Introduction

Intensive and long-lasting research on free-living plant growth-promoting rhizobacteria (PGPR) has emphasized their capacity to improve crop yield and stress tolerance in a more sustainable and environmentally conscious manner. This is thanks to a wide range of PGPR traits, such as atmospheric nitrogen (N) fixation, solubilization of phosphorus (P) and other inorganic nutrients, and the biosynthesis of lytic enzymes, antibiotics, siderophores, and phytohormones, that, as a whole, regulate plant development and promote (a)biotic stress responses [1,2,3]. However, their performance as biofertilizers and biocontrol agents under realistic field and/or greenhouse commercial production conditions has increasingly been reported as inconsistent, limited or even imperceptible. There are many reasons behind their unreliable performance in the field, most resulting from the incompatibility between the controlled laboratory conditions in which they are usually isolated and characterized, and the multifactorial scenarios they will face when used in real-life situations. Failed PGPR performance may be due either to a single factor or to a combination of many factors. These may include the following: physicochemical soil properties (pH, minerals, cation exchange capacity, water and salt content, residual agrochemicals, metal toxicity, etc. [4]); unpredicted rainfall patterns and excessive temperature and light intensity that might interfere with the PGPR’s metabolism and/or with the root’s capacity to expand, among other effects [5,6,7,8]; negative interactions with a competitive local microbiota that may deploy a range of counter-actions, using bacteriocins, physical biofilms, colonizing-inhibiting signaling molecules and other protective strategies to suppress the establishment of the non-native PGPRs [9,10,11]; the absence of beneficial partners; PGPR–plant host antagonism; horizontal genetic exchange; and genetic drift. Other reasons involve the low viability of commercial PGPR formulations, a situation that is exacerbated by prolonged and/or improper storage periods and delayed delivery. An additional factor involves their incorporation in agronomical practices that prioritize optimal crop fertilization and irrigation, a situation that may reduce the nutritional limitations and stress conditions generally needed to facilitate the plant’s rhizosphere colonization by exogenous PGPRs. Finally, an improper choice of phenological stages for the PGPR inoculation of host plants is often made at late vegetative development stages that tend to be the least receptive to PGPR colonization due to the reduced abundance and modified composition of their PGPR-attracting root exudates [12,13,14]. The purpose of this assay is to describe the procedures that our research group implemented in an attempt to realize the full potential of PGPRs in real-world agricultural production settings, using bacterial isolates that were predominantly obtained from the challenging environmental conditions of the semi-desertic regions of Coahuila, located in North–Central Mexico. The reasoning behind this choice was that the harshness of their natural habitat could act as a natural selector of genotypes capable of thriving under highly unfavorable conditions. This narrative will also describe the progress provided by the implementation of some successful strategies for the selection of potentially robust PGPR strains, and also the almost inevitable failures caused by the numerous potential difficulties faced in their application in actual semi-arid field settings.

## 2. Selection of Efficient PGPRs Isolated from Harsh Environments

**Figure 1:**
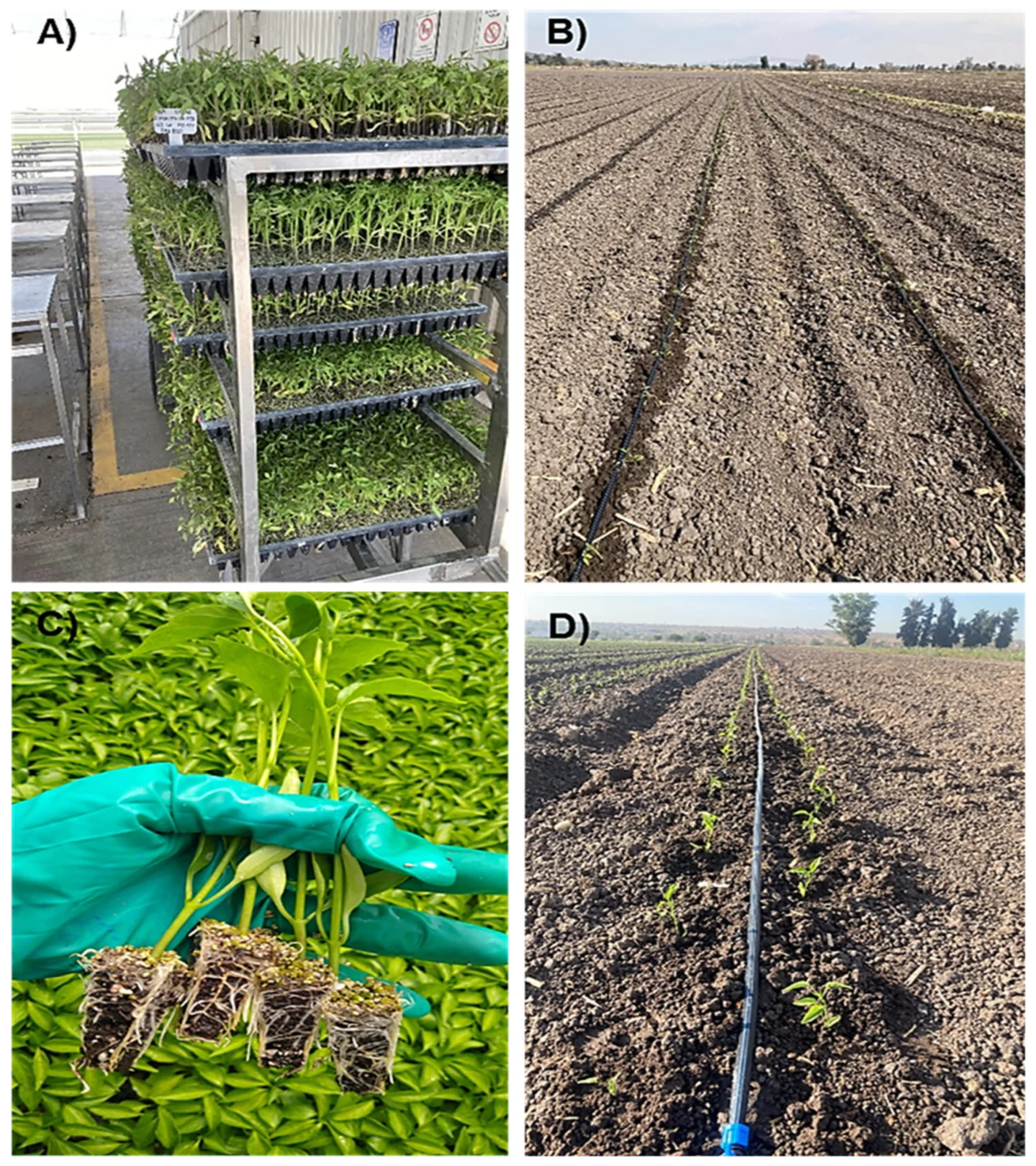
Use of Plant Growth-Promoting Rhizobacteria (PGPRs) for the cultivation of horticultural solanaceous plants in open field conditions. Commercial tomato (*Solanum lycopersicum*) seedlings (DRD8551; SEMINIS) were produced in (**A**) germination trays and (**B**) subsequently transplanted to the experimental field. The same sequence, (**C**,**D**), was used to sow jalapeño chili pepper (*Capsicum annuum*) seedlings (PS11435807; SEMINIS).

Our research group has always avoided the practice of relying solely on in vitro growth-promoting trait analysis for the selection of PGPR isolates. To ensure robustness, this process initiates with the recruitment of specimens from host plants that survive in the stressful living conditions of their native environment or that were artificially forced to sustain them by limiting nutrients and/or through exposure to aggressive soil–plant pathogens. The last option intended to force a stronger and more diverse release of plant exudates able to attract beneficial soil bacteria populations that could help mitigate these unfavorable scenarios [15,16]. This so-called “cry for help” strategy is a normal response used by several plants to palliate negative exposure to (a)biotic stresses [17]. Additionally, the induction of root plant exudates has also been reported to occur in vitro in response to bacterial or fungal elicitors or to chemical inducers of active defense or priming responses [18,19]. Recently, our laboratory employed foliar spraying with benzothiadiazole, a salicylic acid (SA) analog used as a priming-inducing agent [20], or infection with *Clavibacter michiganensis*, an aggressive plant pathogen of solanaceous plants [21], to induce agents on the Micro-Tom and Floradade (*Solanum lycopersicum* L.) commercial tomato varieties. The results were encouraging since a significant increase in the organic acid contents, i.e., citric, malic, and fumaric acids, of their respective root exudates was detected. This result supported the notion that SA signaling regulates the synthesis of root PGPR attractants [22]. This information was taken into consideration in the experimental design of greenhouse and field experiments that resembled the conditions of large-scale agronomic production sites. Hence, field experiments were performed in the summer of 2021 to test the effect of suspending fertilization one week after the application of *Enterobacter* sp. DBA51 or *Bacillus subtilis* LPM1, both of which were isolated from the desertic regions of Coahuila. This procedure was followed by the foliar application of either SA or methyl salicylate (MeSA) three weeks after being transplanted to the soil of tomato (*S. lycopersicum*; DRD8551, SEMINIS) and jalapeño chili peppers (*Capsicum annuum*; PS11435807, SEMINIS) in experimental land plots situated in the state of Guanajuato, México (Figure 1). Here, semi-arid conditions prevail throughout most of the year, except for the rainy season, which extends through the summer and part of the autumn seasons.

**Figure 2:**
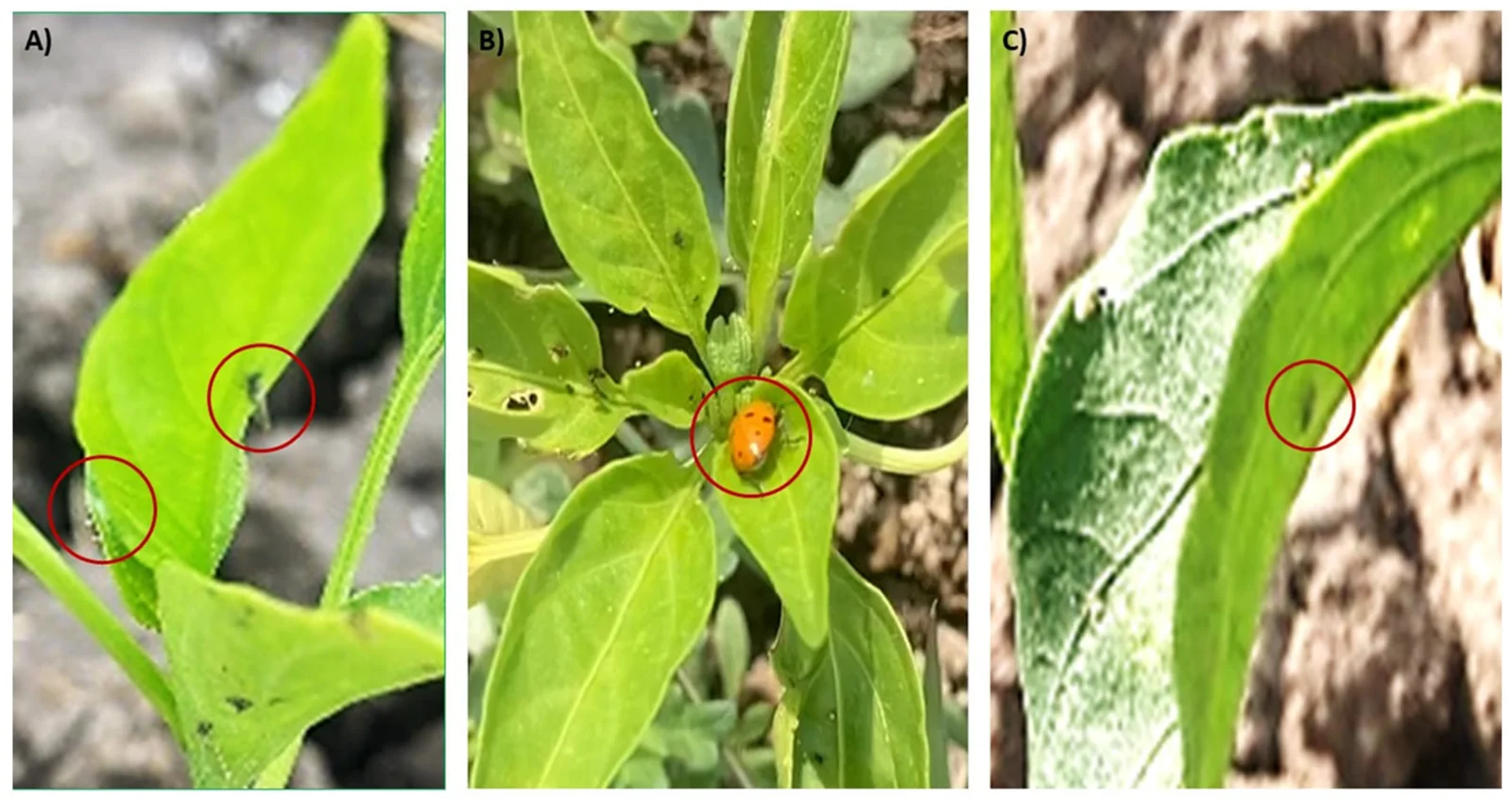
Detection of beneficial insects on the leaves of jalapeño pepper plants (PS11435807). The circles show (**A**) adult specimens and egg masses of *Bactericera cockerelli* Sulc.; (**B**) a ladybug (*Coccinella septempunctata*) feeding on the eggs laid by *B. cockerelli* Sulc. adults, and (**C**) a sighting of an adult lacewing specimen (*Chrysoperla carnea*).

**Figure 3:**
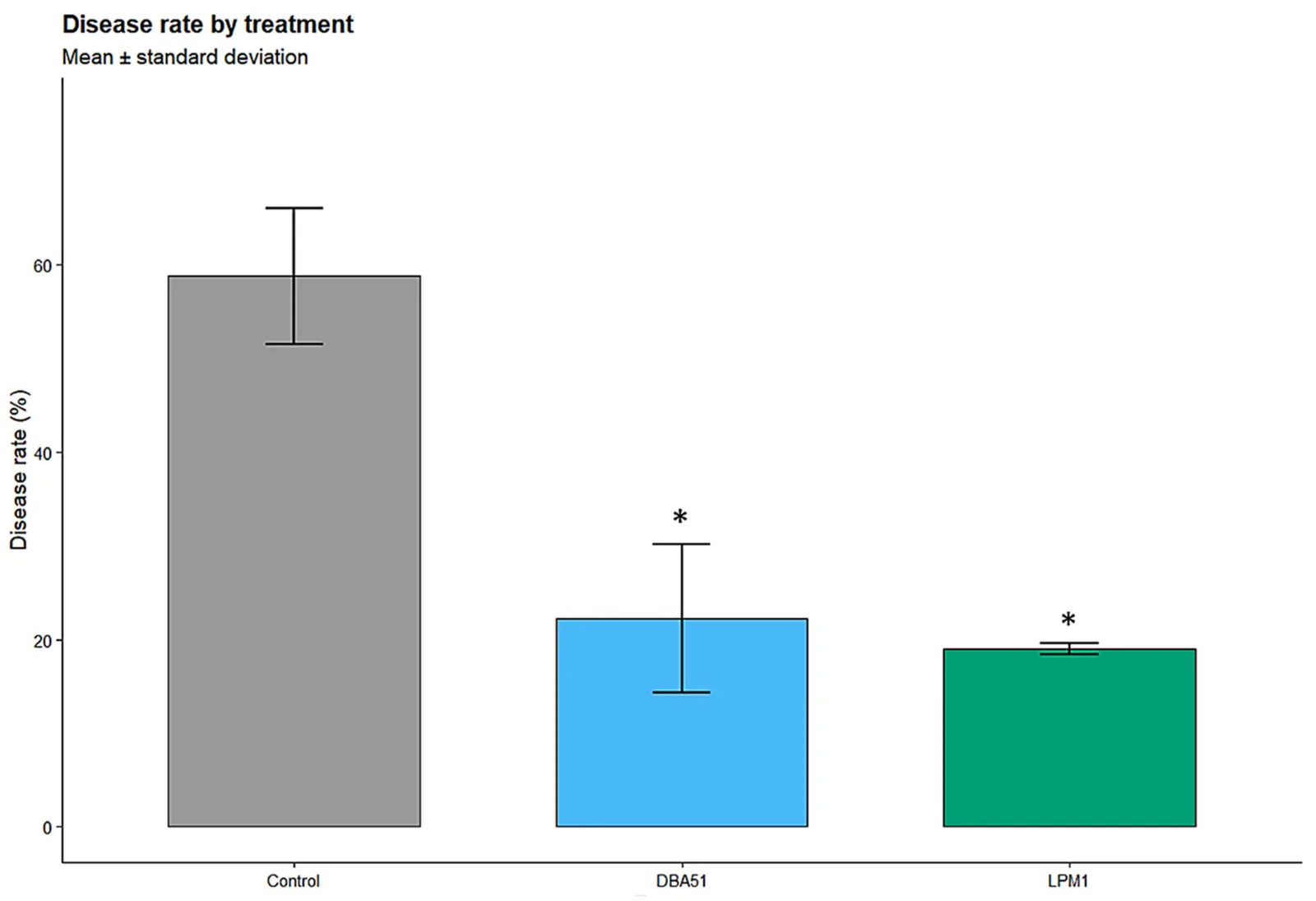
Disease incidence rate in tomato (*S. lycopersicum*, DRD8551) plants inoculated with PGPRs and cultivated under open field conditions. The bars show the percent disease rate ± S.D. in plants inoculated with *Enterobacter* sp. DBA51, *Bacillus subtilis* LPM1 and in uninoculated control plants (figure adapted from data by Délano-Frier et al. [23]). Asterisks indicate statistically significant differences between treatments at *p* < 0.05 using a one-way ANOVA, Tukey HSD test.

The objective of this particular cultivation practice was to improve the interaction of the inoculated bacteria with the plant’s rhizosphere by two means: (i) promoted secretion of root exudates *via* the induction of a nutritional stress post-PGPR inoculation and (ii) complementing this initial stimulus with the subsequent application of SA or MeSA to amplify the systemic activation of root exudates’ secretion [23]. Biometric parameters associated with growth promotion, such as plant height and stem diameter, were significantly higher in treated tomato plants but remained unchanged relative to controls in chili pepper plants. Sightings of beneficial insects such as ladybugs (*Coccinella septempunctata*) feeding on eggs of the potato psyllid *Bactericera cockerelli* Sulc., and of green lacewings (*Chrysoperla carnea*), were frequent (Figure 2). The presence of natural enemies observed in PGPR-treated plants in the field suggests that a potential attracting effect was induced, a possibility that requires further quantitative validation (see below). Additionally, a lower incidence of fungal and bacterial pathogenesis was observed in tomato plants inoculated with the DBA51 and LPM1 PGPR strains (Figure 3). These events suggest the occurrence of various pathogen/insect pest-biocontrol mechanisms, acting alone or in combination, that also requires further research to be clearly defined. A possible scenario involves direct pathogen antagonistic responses, e.g., *via* iron-chelating siderophores, the activated synthesis of defensive secondary metabolites and/or pathogen cell wall-degrading enzymes. [24,25,26]. Others involve parasitism, pathogen toxin inhibition and the elicitation of the induced systemic response (ISR), which enhances the plant’s defensive capacity *via* an SA-independent signaling pathway regulated by jasmonic acid and ethylene [26,27]. Volatile organic compounds from non-pathogenic rhizobacteria could also contribute to the reduction in pathogen incidence and to the observed attraction of predatory insects [28,29].

**Figure 4:**
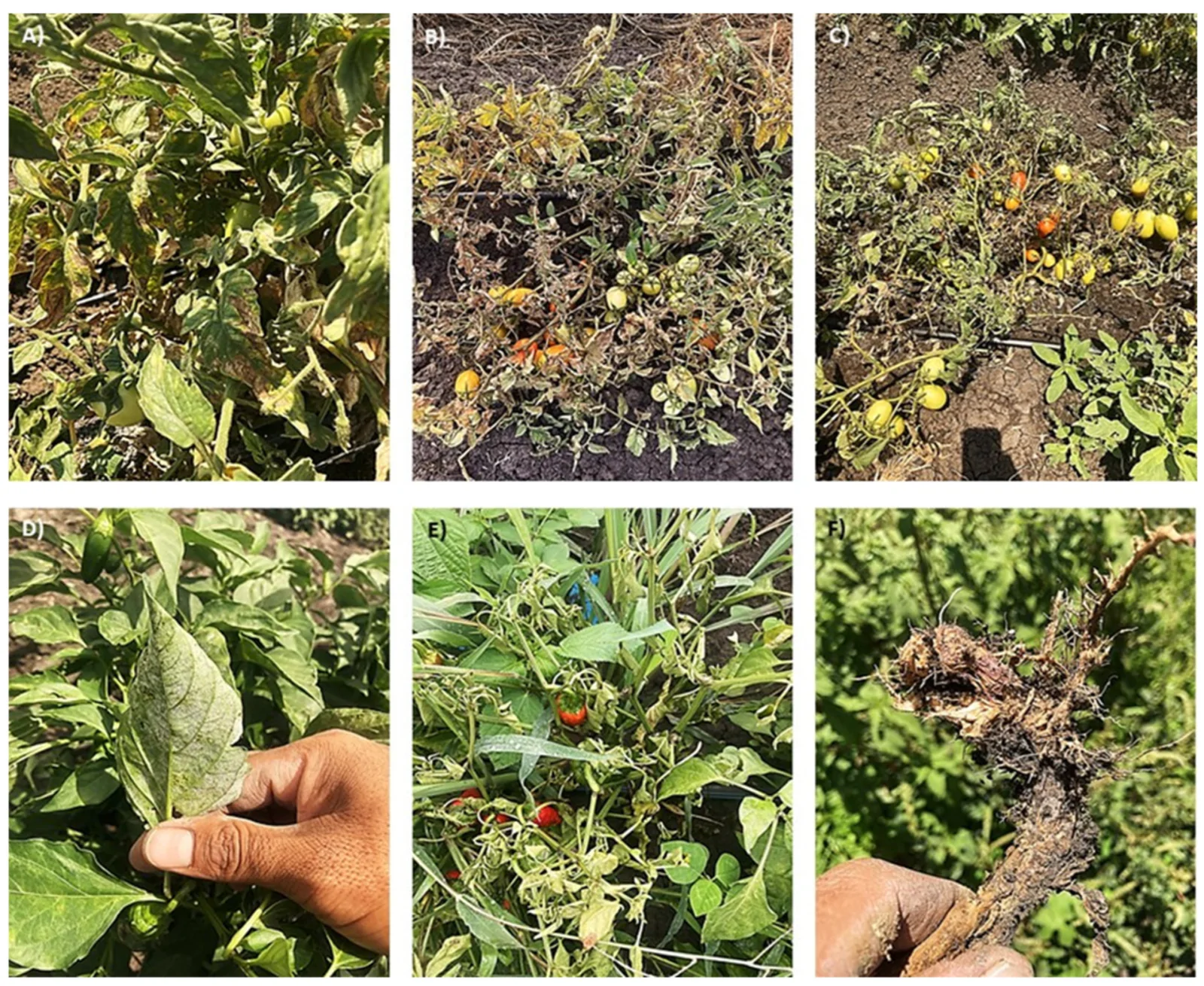
Severity of fungal infection symptoms in field-grown PGPR-inoculated tomato and jalapeño pepper plants. Increased fungal infection damage was observed during late harvesting of tomato (*S. lycopersicum*, DRD8551) and jalapeño pepper plants (*C. annuum*, PS11435807) inoculated with either *Enterobacter* sp. DBA51 or *Bacillus subtilis* LPM1. The images show symptoms of (**A**) late blight and (**B**,**C**) vascular wilt in tomato, and of (**D**) powdery mildew, (**E**) vascular wilt, and (**F**) extensive root rot in jalapeño plants.

An unexpected factor that affected the yield of both crops was a 45-day delay in the transplantation of the nursery-germinated seedlings to the soil. The unforeseen consequences were a reduced time-window available for the harvesting cycles (only three could be performed) due to the entry into the heaviest precipitation period of the rainy season. Difficult weather impeded access to the fields for several days and also promoted the appearance of fungal- and oomycete-caused diseases such as late blight, vascular wilt, powdery mildew, and others (Figure 4). Despite this contingency, crop yields in treated jalapeño pepper plants significantly increased, ranging from 75 to 80% higher than control plants.

## 3. Applicability of Desert Environment PGPRs in Alternative Cultivation Practices, Such as the Use of Shading and/or Plastic Mulches in Intensive Agriculture

The use of plastic mulches in agriculture is a common practice that provides several benefits to the crops, such as preventing weed growth, maintaining plant growth-promoting soil temperatures, and decreasing the evapotranspiration of water from the soil [30]. Their use, together with PGPRs and other beneficial microbes, has been reported to increase the efficiency and reduce the consumption of resources, respectively, lessen the damage caused by abiotic stress exposure, improve weed and disease control, facilitate the plant–soil–microorganism tripartite relationship, and stimulate the growth, yield, and/or final quality of commercial crops [31]. Hence, mulching with clear polyethylene and other less-used synthetic polymer- and biodegradable material-based films [32,33] has become a common technique to promote growth in rain-fed regions prone to insufficient precipitation and high temperatures. However, their use has raised concerns regarding the potential damaging effects on the environment, primarily caused by soil and water pollution derived from the accumulation of plastic residues and microparticles (MPs) released by weathered films and higher carbon (C) footprints. In this respect, recent two-year field studies conducted mostly on wheat and under rain-fed conditions compared synthetic and biodegradable film mulching in terms of socio-economic and environmental benefits. One concluded that both mulching film alternatives improved the soil’s hydrothermal conditions, increased yields, and enhanced net returns. However, biodegradable mulches tended to have lower C footprints, mostly by improving soil organic carbon sequestration [34]. Two others obtained similar results using clear polyethylene and black synthetic mulching, which either promoted soil C sequestration [35], or positively influenced N use efficiency and net ecosystem economic budget in addition to maintaining adequate C sequestration rates [36]. A cabbage production field study also reported that the use of biodegradable black mulch reduced CO_2_ and N_2_O emissions, enhanced the complexity and stability of the soil bacterial network and improved several soil physicochemical properties [37]. In sharp contrast, a large meta-analysis revealed that mulching did, in fact, lead to the accumulation of MPs in the soil. These were found to negatively affect plant growth and development, disrupt soil function and structure, modify the levels of dissolved organic C and N and perturb N cycling. Microbial diversity and soil fauna were also decreased or damaged [38].

Both contradictory scenarios strongly suggest that future research should concentrate on the performance of more long-term field studies done under varying climatic situations in order to develop self-remediating agroecosystem alternatives to conventional plastic mulching [39]. A promising development in this direction has been the development of improved biodegradable mulching films that do not accumulate environmental waste [40]. Other promising alternatives involve the use plant-based strategies to reduce MP pollution in agricultural ecosystems based on bioaugmentation with plastic-degrading microbial consortia, inoculation with rhizospheric microbes able to protect against MP-related stress, and/or the use of organic amendments, e.g., biochar, to reduce MPs’ toxicity [41].

**Figure 5:**
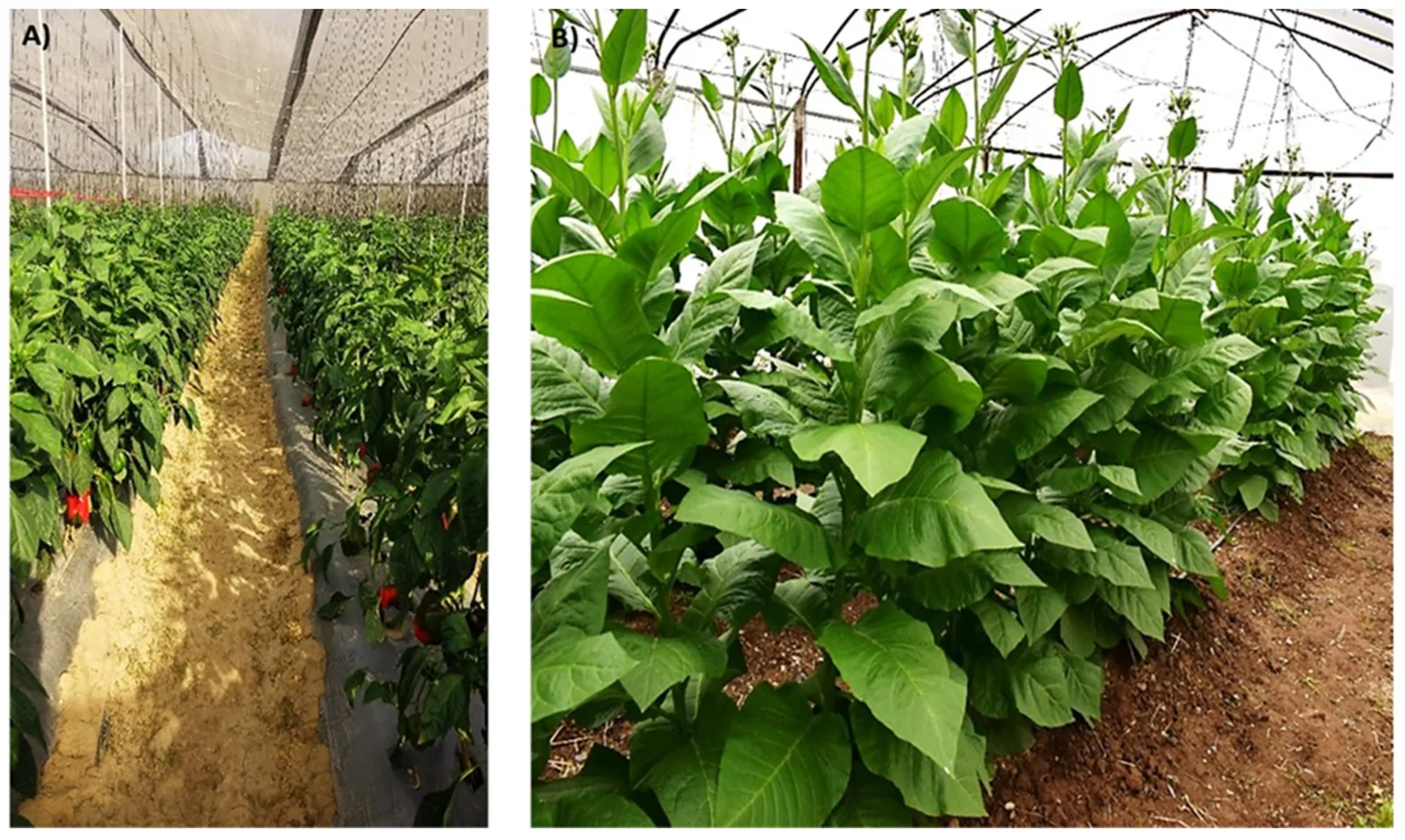
Use of PGPR in plants cultivated under shading conditions ± the use of plastic mulching. (**A**) Cultivation of commercial bell pepper (*Capsicum annuum* L.) inoculated with *Bacillus subtilis* LPM1 grown using shade and plastic mulch films; (**B**) fully grown tobacco (*Nicotiana tabacum* L.) shade-maintained plants inoculated with *Enterobacter* sp. DBA51.

In this context, further experiments were performed to test the potential advantages offered by the employment of synthetic black soil mulching and PGPRs for the cultivation, under shading, of three different commercial varieties of bell pepper plants, i.e., *Prosperity RZ*, *Valkira RZ* and *Sven RZ*, inoculated with *B. subtilis* LPM1 (Figure 5A). LPM1 significantly promoted vegetative growth, as evidenced by the wider stems, augmented total dry biomass and more efficient photosynthetic CO_2_ assimilation rates measured 60 days after PGPR inoculation in the three varieties [42]. However, these promising growth-promotion effects did not translate into higher yields, although a tendency towards a reduced incidence of leaf powdery mildew was observed. This observation suggested the activation of a PGPR-induced systemic defense response (see above), which was unfortunately not documented. Additional information regarding the way mulching and the application of a non-native PGPR affected the soil’s native microbial community, as mentioned previously, was also lacking. Gathering information able to shed light on the cause(s) of the effects observed in these trials will be a major objective in future experimentation.

Nevertheless, the missing link between growth promotion and yield observed in the chili pepper plants cultivated under the above conditions resembled the results of a recent study using cotton as the experimental model. Here, the enhanced underground growth facilitated by the use of black plastic mulch under well-irrigated conditions was not reflected by an increase in seed and lint cotton yields and quality. Likewise, the significant increases in stem diameter, CO_2_ assimilation, boll number and tolerance to water-deficit stress obtained when PGPRs were added as an additional experimental factor did not translate into higher yields or better quality [43]. The above effects were in agreement with the long-standing concept that the C input from photosynthesis is unable, *per se*, to increase grain crop yields, contrary to the critical role played by the availability of water and other nutrients, predominantly N [44]. Surprisingly, abscisic acid was recently proposed to participate in various plant developmental events and to be a positive contributor to enhanced yields, particularly when maintained at basal levels [45]. Other possible circumstances proposed to disconnect growth promotion from augmented crop yields include: (i) an inefficient partitioning of sugars and nutrients into sink organs; (ii) hormonal overstimulation by PGPR, which may divert the plant’s metabolism toward rapid cell elongation and vegetative vigor and away from reproductive development; and (iii) poor soil colonization due to competing native soil microbiota [46,47,48].

Further experiments involved shade-cultivation of tobacco plants inoculated with *Enterobacter* sp. DBA51 (Figure 5B). Regular monitoring of the plants during their development to maturity indicated that PGPR inoculation only had a noticeable effect on the root biomass, which was significantly higher than that of the untreated controls. Another remarkable observation was that the PGPR-inoculated plants showed no symptoms of foliar or root diseases, nor were they damaged by folivorous insects, and that the tobacco responses to PGPR inoculation differed from those observed in other similarly treated solanaceous plants (unpublished results). The observed differences were most probably a reflection of the variations in the chemical composition of root exudates, which are known to influence the dynamic interaction between PGPRs and the rhizosphere in a very specific way. This interaction is primarily driven by plant-specific mixtures of root-derived metabolites, mainly sugars, amino and organic acids, phenolic compounds, fatty acids and sterols. These vital chemicals function together with signaling molecules as strong chemo-attractants and nutrient sources that selectively recruit and sustain specific microbial communities from the bulk soil [49,50]. An additional possible reason for the diverging growth-promoting effects observed in the model solanaceous plants is that they resulted from the utilization of different growth-promoting mechanisms [51].

**Figure 6:**
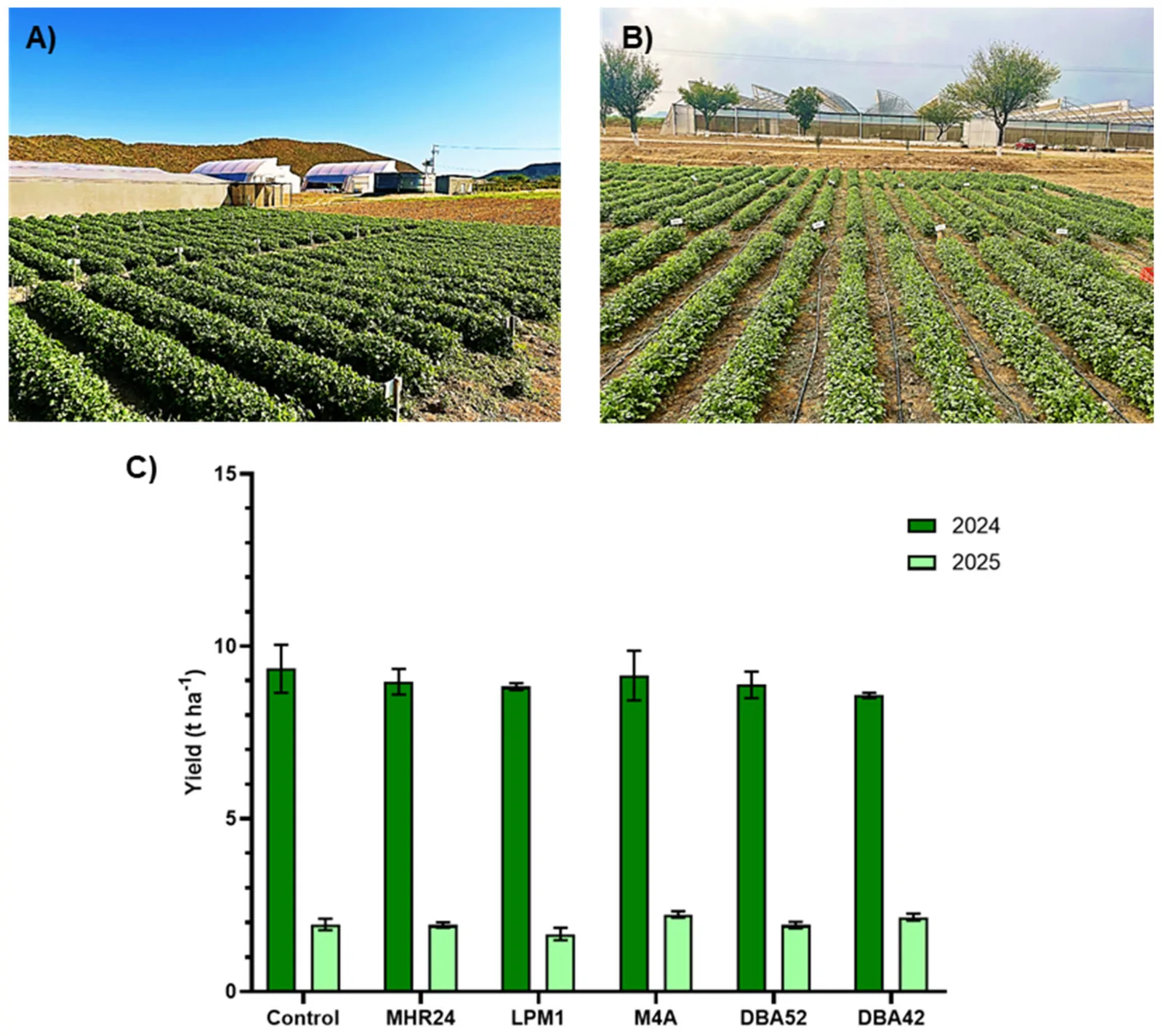
Open-field cultivation of parsley (*Coriandrum sativum* L.) plants inoculated with PGPRs isolated from the semi-desertic region of Coahuila, Mexico. Panels (**A**,**B**) show a section of the experimental fields employed to cultivate parsley plants inoculated with these desert-dwelling PGPRs during October and November of 2024 and 2025, respectively. Panel (**C**) shows the yields obtained in each agricultural cycle by the inoculated parsley plants. The PGPRs tested were MHR24, *Bacillus amyloliquefaciens* MHR24; LPM1, *B. subtilis* LPM1; M4A, *Serratia marcescens* M4A; DBA42, *Enterobacter hormaechei* DBA42; and DBA52, *Enterobacter soli* DBA52.

A lack of reproducibility was also observed when five different PGPRs isolated from the semi-desert lands of Coahuila were used to inoculate parsley (*Coriandrum sativum* L.), a short-term crop usually harvested 50 days after sowing, during the autumn–winter cycles of 2024 and 2025. The yield obtained in the first year was ca. 35 kg per treatment (Figure 6A,C), whereas in the second year it averaged 6–9 kg per treatment (Figure 6B,C). The discrepancy occurred despite the fact that the experiments were performed using the same bacterial titer (1 × 10^7^ CFU/ml), plant population density, and fertilization regime, and that the climatic conditions in both years were fairly similar. Also, no incidence of disease or insect infestation damage was observed in both years, except for the need for an additional weeding procedure in the year 2025. However, prior to this series of experiments, the experimental parcels remained mostly fallow, and were only used to cultivate tomato plants in the spring–summer cycle of 2025, prior to the second parsley cultivation trial. It is proposed that this intermediate cropping event could have modified the nutritional and/or microbiological status of the soil, and thereby the performance of the PGPRs that were subsequently used. It also remains uncertain whether the beneficial effects of PGPR treatments can be fully realized in short-term cultivation periods.

These non-reproducible and/or underperforming PGPRs results in realistic agronomic production scenarios agree with numerous previous studies that reported on the inconsistency of PGPR’s efficiency in the field. As mentioned above, the climate may be an important factor, since climatic differences are known to influence PGPRs’ impact by altering the diversity and function of plant-associated microbial communities [52,53]. A relevant example of the climatic effect was the variable response of wheat crops to PGPR inoculation observed in the Punjab region of South Asia, where several different ecological conditions are prevalent [54]. Climatic variations might also produce changes in plant physiology and root exudation, as demonstrated by a study in which the presence of L-tryptophan in root exudates decreased in response to climatic oscillations, which lowered auxin production by the soil microflora and introduced PGPRs alike, and, thereby, to a reduction in growth-promoting activity [55]. Temperature variations have also been observed to change the composition, abundance or activity of rhizosphere-associated microbial communities, and their colonization capacity [52]. In addition, and similarly to our experience, other workers have found that different plant species, and even varying plant cultivars of the same species, can establish unique relationships with PGPRs that significantly impact their field performance [56].

Finally, the presumed enrichment of N and P that occurred in the soil fertilized to cultivate tomato plants in the growing cycle prior to the second parsley experiment may have been enough to create a stressful ambient for the PGPRs that were later employed, leading to reduced physiological and enzymatic capabilities [8]. In this respect, a short-term application of urea was shown to significantly change the soil microbial community and to disrupt the activity of complex substrate-degrading extracellular microbial enzymes [57,58]. Additionally, lentil field trials that employed growth regulators and PGPR inoculation under dryland conditions over two consecutive years revealed complex and difficult-to-define interactions between yield components and physiological traits. Thus, further research is still needed to determine crop performance in rotations with greater precision [59].

Altered soil respiration, leading to lower C storage, and soil organic matter were also found to be contributing factors to inter- and intra-annual fluctuations in crop performance, as described by a 2-year field trial study performed in the semi-arid conditions of the Chihuahuan desert [60]. Additional crucial observations were the lower crop performance in agricultural sites compared to xeric shrubland lands and the higher sensitivity of the former to soil temperature changes. These differences were attributed, in part, to management practices, such as tillage and fertilizer use, which decreased soil C concomitantly with soil organic matter decomposition [60]. In this respect, a previous study also found that increased soil respiration could be caused by an excessive soil tilling, which exposed stored soil organic matter and accelerated its metabolism. This chain of effects usually led to degraded soils having reduced microbial diversity [11,12,61,62,63]. Moreover, soil microbial communities from high-intensity land use were found to have a lower ability to adapt to environmental stress conditions [64]. In this respect, a no-tillage regimen in semi-arid agricultural soils was reported to significantly benefit the soil bacterial community, particularly the photosynthetic organisms involved in the formation of biological soil crusts. The benefit of this layer to semi-arid agricultural systems is significant, since it reduces soil wind erosion and contributes to its stabilization and water-holding capacity [65].

## 4. Conclusions and Points to Ponder

The results obtained so far from our use of PGPR in agricultural production scenarios indicate that several aspects have to be considered to ensure that the desired positive effects in terms of plant growth and yield are produced:

(1) It is indispensable to know the physicochemical characteristics of the water and soil used for irrigation and cultivation, respectively.
(2) Information about the microbiological composition of the soil and its fluctuations with time and seasonal changes is fundamental. It is recommended to obtain information on the performance of field experiments in different climates and soil types and on different crops to gain a clear perspective of the performance of isolated PGPRs under diverse natural conditions.
(3) The use of PGPRs isolated from harsh environments with the ability to modulate their relationship according to the chemical context of the rhizospheres of different crop plants might yield better results. However, rigorous further efforts should be made to confirm the ability of PGPRs isolated from challenging habitats to function in response to high acidity/alkalinity, desiccation, and fluctuations in temperature, salinity, and other aspects.
(4) The fluctuating results described here suggest that the application of a combination of PGPR strains as a consortium, which could include other plant growth-promoting microorganisms (PGPMs) such as mycorrhizal fungi, might have been a more advantageous practice, as higher microbial diversity usually leads to a stronger symbiotic relationship with the host plants. Other future research prospects include the use of emerging strategies, based on state-of-the-art biotechnological procedures, to significantly enhance the efficiency and selectivity of tailored plant–PGPR interactions. The feasibility of several novel developments has already been demonstrated by works describing the use of multi-omic and related bio-informatic data to guide the generation of improved or synthetic versions of PGPR, either individually or as consortia. The latter, colloquially known as SynComs, have been reported to enhance root colonization abilities, strengthen stress-ameliorating capacities, heighten tolerance to toxic soil conditions and improve the interaction with host plants. They can also provide an improved capacity to modulate root exudate composition, a property known as host-mediated microbiome engineering. Additional achievements involve facilitating the formulation of precise bioinoculants and the development of nano-biofertilizers [66,67,68].
(5) The results also confirmed that different plants produce different root exudates that may influence PGPR behavior in distinct manners and, thereby, generate diverse responses to the microbial inoculants. This leads to the recommendation that PGPR inoculants should be plant cultivar-specific in order to ensure the establishment of the most productive plant–PGPR association.
(6) Additional key factors to be considered are the need for proper awareness of the optimal periods to start the cultivation of a given crop and its precise nutritional needs and pathogen/pest defensive capabilities, and to select the best developmental stage and/or priming treatment to encourage the best possible response to PGPR inoculation. Moreover, constant vigilance should be exerted to detect and/or avoid the misuse of synthetic fertilizers, leading to the unwanted enrichment of nutrients, primarily N and P, which could negatively affect PGPR performance.
(7) Finally, and as mentioned in this communication, several of the methods employed to improve agricultural production in the present day are not devoid of limitations. This situation has stimulated the gradual emergence of several other green technologies that have the potential to greatly improve agricultural production. These are based on the application of computer vision technology or the use of diverse physical technologies, in addition to nanotechnology and intelligent field management. The latter is a tool that produces rapid and precise analyses of plant phenotypes that also greatly increases the capacity to control plant diseases and the optimal regulation of seed germination and plant growth [69]. Our group is particularly focused on the development of nano-biofertilizers, which entails generating nano-particle-coated PGPRs and/or other PGPMs. We believe that such modifications will effectively protect them from harsh environmental conditions, significantly extend their shelf life and permit their controlled release in soil niches, without impacting PGPMs’ growth, viability, and functionality, or their stress-ameliorating mechanisms and plant growth- and yield-promoting capabilities. The candidate PGPR strains for this endeavor are *Bacillus amyloliquefaciens* MHR24, *Serratia marcescens* M4A, and *Enterobacter hormaechei* DBA42, which have consistently shown the best performance in tomato and jalapeño pepper crops under open-field conditions. The MHR24 and M4A strains exerted a biocontrol effect against fungal phytopathogens while promoting growth and increased yields. On the other hand, the M4A and DBA42 strains synthesized 1-aminocyclopropane-1-carboxylate deaminase activity and indol acetic acid, which are important traits associated with water-deficit stress tolerance, in addition to possessing growth-promotion activity and yield-increase potential. Another promising objective is to use synthetic biology tools to optimize the biological N fixation of root-associated diazotrophic bacteria in order to reduce the use of synthetic N fertilizers in agronomic production schemes [70,71].

## Data Availability

The original contributions presented in this study are included in the article. Further inquiries can be directed to the corresponding author.
